# Responsiveness of a modified version of the postural assessment scale for stroke patients and longitudinal change in postural control after stroke- Postural Stroke Study in Gothenburg (POSTGOT) -

**DOI:** 10.1186/1743-0003-10-8

**Published:** 2013-01-29

**Authors:** Carina U Persson, Katharina S Sunnerhagen, Anna Danielsson, Anna Grimby-Ekman, Per-Olof Hansson

**Affiliations:** 1 The Institute of Neuroscience and Physiology, Department of Clinical Neuroscience and Rehabilitation, Sahlgrenska Academy at the University of Gothenburg, Göteborg, Sweden; 2 Sunnaas Rehabilitation Hospital, the Medical Faculty, Oslo University, Oslo, Norway; 3 Occupational and Environmental Medicine, Sahlgrenska University Hospital, University of Gothenburg, Göteborg, Sweden; 4 The Institute of Medicine, Sahlgrenska Academy at the University of Gothenburg, Göteborg, Sweden

**Keywords:** Postural Balance, Clinometric, Recovery of function, Outcome

## Abstract

**Background:**

Responsiveness data certify that a change in a measurement output represents a real change, not a measurement error or biological variability. The objective was to evaluate the responsiveness of the modified version of the Postural Assessment Scale for Stroke Patients (SwePASS) in patients with a first event of stroke. An additional aim was to estimate the change in postural control during the first 12 months after stroke onset.

**Methods:**

The SwePASS assessments were conducted during the first week and 3, 6 and 12 months after stroke in 90 patients. Svensson’s method, Relative Position (RP), Relative Concentration (RC) and Relative Rank Variance (RV), were used to estimate the scale’s responsiveness and the patients’ change in postural control over time.

**Results:**

From the first week to 3 months after stroke, the patients improved in terms of postural control with 2 to 12 times larger systematic changes in Relative Position (RP), for which 9 items and the total score showed a significant responsiveness to change when compared to the intrarater reliability measurement error of the SwePASS reported in a previous study. When SwePASS was used to assess change in postural control between the first week and 3 months, 74% of the patients received higher scores while 10% received lower scores, RP 0.31 (95% CI 0.219-0.402). The corresponding figures between 3 and 6 and between 6 and 12 months were 37% and 16%, RP 0.09 (95% CI 0.030-0.152), and 18% and 26%, RP −0.07 (95% CI −0.134- (−0.010)), respectively.

**Conclusions:**

The SwePASS is responsive to change. Postural control evaluated using the SwePASS showed an improvement during the first 6 months after stroke. The measurement property, in the form of responsiveness, shows that the SwePASS scoring method can be considered for use in rehabilitation when assessing postural control in patients after stroke, especially during the first 3 months.

## Background

The consequences of stroke often include impaired postural control [1]. Several clinical scales and tests are used to assess postural control. In order to make a meaningful interpretation of the results of the clinical scales and tests used, it is fundamental that the tests are reliable and valid and that they are able to detect changes, which is defined as responsiveness [2]. In clinical practice, even a small change, either an improvement or deterioration, can make a difference in terms of further treatment. Responsiveness data certify that a change in a measurement output represents a real change, not a measurement error or biological variability [3].

There is no gold standard for assessing responsiveness [2,4]. However, it is fundamental that the clinical status is expected to change. Furthermore, criteria are needed to identify whether patients have improved or deteriorated in function/activity over time [2]. The criteria may be anchor based (such as clinician or patient-rated improvements) [5] or distribution based (relying on statistical properties), or both, and should be obtained in a population using a longitudinal approach [6].

There are several assessment scales for postural control [7-11]. The French Postural Assessment Scale for Stroke Patients (PASS) [11], derived from the Fugl-Meyer assessment of balance and mobility [9] and the Birgitta Lindmark Motor Assessment [10], was originally created to assess postural control in patients with stroke. Compared to the Berg balance scale [7,8], the most frequently used clinical scale for assessing postural control in the elderly, the PASS, in addition to having stroke as a specific target group, also comprises a patient’s capacity to roll in a lying position. SwePASS was developed and modified from the original PASS [11]. In order to further improve the clinical usability of the scale, SwePASS has changes in the definitions for the item categories. In SwePASS, score 1 is defined as “with support from 2 persons” instead of “with much help” as in PASS. The definition for score 2 is defined in SwePASS as “with support from 1 person” instead of “with little help” in PASS. Furthermore, to focus more on the vertical movement than on fine motor skills, in SwePASS the patient is “picking up a shoe from the floor” instead of “a pencil” in PASS. In addition, “arm movement over shoulder level” is defined in SwePASS as “pull hand/s from forehead to neck (as pull fingers through the hair) altered with arm/s hanging parallel with the trunk to avoid tiredness). The Swedish modified version of the Postural Assessment Scale for Stroke Patients (SwePASS) has previously been shown to be a highly reliable (with high interrater and intrarater reliability) clinical scale, easy to apply and fast to administer, in patients with acute stroke [12] as well as a moderate predictor of the risk of falling after stroke [13].

Two previous studies showed that the original Postural Assessment Scale for Stroke Patients (PASS) was sensitive for assessments of stroke survivors during the first 3 months after the stroke, but, probably due to a ceiling effect, was found to be less sensitive thereafter [11,14]. PASS was recently found to have a high internal responsiveness in patients with stroke [15]. However, no previous studies have been done on the responsiveness to change of SwePASS.

Earlier studies have found that recovery after stroke shows a non-linear pattern over time [16-18], with the greatest improvements during the first months after stroke [19]. It is unclear whether significant improvements in body function and activity occur after that [18,20,21]. There is a gap in knowledge about responsiveness in SwePASS and recovery in postural control after stroke, and further longitudinal studies are thus needed [22]. The aim of the present study was to investigate the responsiveness of SwePASS in patients with a first event of stroke during the first 3 months after stroke, assuming an improvement during this period [18]. An additional objective was to use the SwePASS to estimate the longitudinal change in postural control during the first 12 months after stroke.

## Methods

### Participants

The participants in the present study were a subsample of the 96 patients in the follow-up part of the Postural Stroke Study in Gothenburg, POSTGOT [12], and consist of these 90 patients who participated in at least two consecutive follow-up occasions after discharge during the first year after stroke. POSTGOT consists of patients admitted to a stroke unit [12,13]. The inclusion criterion was a first-ever stroke, defined according to World Health Organization criteria [23]. Exclusion criteria were co-morbidities such as leg amputation, a diagnosis of dementia or severe psychiatric diseases that could interfere with postural control and the ability to cooperate during the assessments. Ischaemic stroke events were classified according to the Trial of Org 10172 in Acute Stroke Treatment (TOAST criteria) [24]. The Regional Ethics Committee of Gothenburg approved the study, and informed written consent was obtained. Data on recurrent stroke during follow-up were collected from medical journals, and assessments made after a second stroke were excluded from the calculations.

The participants in the study had a median length of stay of 14 days (range: 4–79 days). At the stroke unit, physiotherapy and occupational therapy was offered 5 days a week. Of the 90 patients who participated in at least 2 consecutive follow-up assessments, 75 patients (83%) were discharged to their own home, 12 to a nursing home, 2 to a geriatric rehabilitation clinic and 1 to a rehabilitation medicine clinic.

A needs-based rehabilitation was offered according to the Swedish healthcare system. At discharge, 57 patients (63%) were assessed as having no further need of rehabilitation. The rehabilitation plans for the remaining 33 patients (37%) were as follows: outpatient rehabilitation for 12 patients, primary care rehabilitation for 7, home healthcare for 4, primary care rehabilitation and outpatient rehabilitation for 1 patient, referral to a rehabilitation medicine clinic for 1 patient and “other rehabilitation services” for 8 patients.

### Outcome measure

SwePASS is an ordinal scale with 12 items, scored from 0 to 3, concerning the maintenance of a given posture in the sitting and standing positions and the equilibrium in changing positions in lying, sitting and standing. The scale was developed from the original Postural Assessment Scale for Stroke Patients (PASS) [11] with some minor modifications in order to further improve the clinical usability of this scale. The 12 items are the following: supine to affected side lateral, supine to non affected side lateral, supine to sitting on the edge of bed, sitting without support, sitting to standing up, standing with support, standing without support, standing on the non paretic leg, standing on the paretic leg, standing, picking up a shoe from the floor, sitting down from standing up and sitting on the edge of bed to supine. The item scores are summed up to a total score, with a maximum of 36. Higher scores indicate better postural control.

### Procedures

To estimate changes in postural control during the first year, the patients were examined with SwePASS on 4 different occasions. The first assessment was made during the first week after stroke onset (between days 4 and 7, median day 5) in the patient’s room at the stroke unit by a physiotherapist who was not involved in the rehabilitation. The patients were followed up with a second, third and fourth assessment at 3, 6 and 12 months after stroke, respectively. At the follow-up investigations, the patients came to the stroke unit and the SwePASS was carried out in the training room. All the physiotherapists involved were instructed and trained in how to perform the assessments before their involvement in the study. At the follow-ups, the aim was to have the same physiotherapist perform the assessments, which was achieved in 44% of the assessments. For the follow-up investigations, a time window of ±14 days was allowed.

### Statistics

The Statistical Package for Social Services (SPSS^©^) (Version 17 SPSS Inc., Chicago, IL, USA) was used for basic statistics. Responsiveness calculations were based on assessments at the first week and 3 months after stroke, using one pair of a single group before and after design [6]. Change over time calculations were based on assessments at the first week and 3, 6 and 12 months after stroke onset. Svensson’s rank-invariant method for ordinal data was applied to estimate responsiveness and change over time [25] using a specifically programmed Excel file [26]. Svensson’s method estimates the systematic differences between the assessments, relative position (RP) and concentrations of the score chosen, relative concentration (RC). Furthermore, the relative rank variance (RV) was used to estimate individual non-systematic changes in the paired ordinal data [25,26]. The RP and RC are given values from −1 to 1, while the RV is given values from 0 to 1. A higher RP value indicates a greater change between assessments. A higher RC value indicates a greater concentration towards central categories. High RV values indicate individual variation and, hence, heterogeneity in the group. Patients who received the maximal SwePASS score at the first assessment were excluded from the responsiveness calculations. A difference was assumed to be a real change if the confidence intervals (CIs) for the RP, the RC and the RV did not overlap the corresponding CIs from a reliability study [12], as those indicate the precision of the assessments. The change in the SwePASS over the first 12 months was regarded as statistically significant at the level of α=0.05 if the 95% CIs for the RP and/or the RC did not include null. A Mann–Whitney U Test was performed to evaluate whether there was any statistically significant difference in outcome between those who were referred to training and those who were not.

## Results

The characteristics and the results of the clinical assessment scales at baseline for the patients who participated in the three different time periods are shown in Table 1.

**Table 1:** Characteristics and results from the Berg Balance Scale (BBS) and the Modified Motor Assessment Scale (M-MAS UAS-95) at baseline

	**Participants during the period;**		
	**0 to 3 months**	**3 to 6 months**	**6 to 12 months**
**Characteristics**	**n=72**	**n=71**	**n=65**
Age, years, median (range)	73 (50–94)	73 (47–92)	73 (47–90)
Patients, n (%)			
Female	33 (46)	27 (38)	26 (40)
Male	39 (54)	44 (62)	39 (60)
Stroke classification (TOAST), n (%)			
Large vessel disease	17 (24)	18 (25)	17 (26)
Small vessel disease	21 (29)	21 (30)	17 (26)
Cardioembolic stroke	15 (21)	11 (15)	11 (17)
Cryptogenic stroke	13 (18)	14 (20)	12 (19)
Intracerebral haemorrhage	6 (8)	7 (10)	8 (12)
Side of lesion, n (%)			
Right side lesion	35 (49)	32 (45)	28 (43)
Left side lesion	37 (51)	39 (55)	37 (57)
Hypertension	47 (65)	44 (62)	41 (63)
Diabetes mellitus	17 (24)	18 (25)	17 (26)
Results from clinical scales 1–7 days after stroke onset			
BBS median (range) (n)	35 (0–56) (n=71)	41 (0–56) (n=70)	41 (0–56) (n=64)
M-MAS UAS-95 median (range)	45 (12–55) (n=65)	47 (12–55) (n=65)	50 (16–55) (n=59)

### Responsiveness

**Figure 1:**
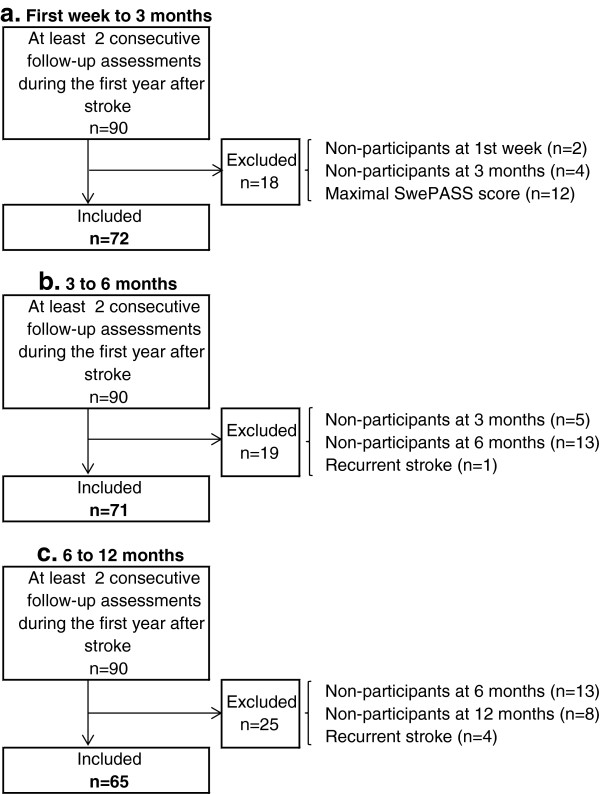
The flow chart for the inclusion of patients in the estimations of responsiveness and the change over time.

These results are based on the 72 patients who participated in the assessments during the first week and at 3 months after stroke. Figure 1 shows the flow chart for the inclusion of patients in the estimations of responsiveness and the change over time. Improved postural control (higher SwePASS total scores) was observed in 53 of 72 patients (73%) from the first week to 3 months after stroke, while deterioration was seen in 7 patients (10%) and an unchanged score in 12 patients (17%). The median total SwePASS score was 30 (min-max: 6–35) during the first week and 31 (min-max: 10–36) at 3 months. The mean SwePASS total score was 28 (SD 7.1) during the first week and 31 (SD 5.0) (n 72) at 3 months.

Table 2 shows that all items and the total SwePASS score had systematic changes in RP that were 2 to 12 times larger compared with the reference values from a previous study of intrarater reliability [12]. Nine items and the total score showed statistically significant responsiveness from the first week to 3 months. The largest systematic change was found for the total score (RP 0.31). Item 9 showed a statistically significant systematic change in the concentration of scores (RC −0.24, 95% CI −0.39 to −0.08). No other item showed any systematic changes in concentration (RC values between −0.14 and 0.03). Concerning relative rank variance, item 8 (RV 0.04, 95% CI 0.00-0.072), item 9 (RV 0.06, 95% CI 0.006-0.126) and the total SwePASS score (RV 0.13, 95% CI 0.034-0.232) showed statistically significant values. All other items had RV values of zero or close to zero (RV values between 0.000 and 0.002).

**Table 2:** Responsiveness of the SwePASS based on systematic changes in relative position from the first week to 3 months after stroke (n=72) compared to reference values of the intrarater reliability measurement error for the SwePASS

	**First week to 3 months**			*Reference values			**Responsiveness**
	**95% CI**			**95% CI**			
**Item**	**RP**	**Lower**	**Upper**	**RP**	**Lower**	**Upper**	
1. Supine to affected lateral side	0.07	0.001	0.140	0.03	0.001	0.066	No
2. Supine to non-affected lateral side	0.09	0.012	0.160	0.01	−0.001	0.025	No
3. Supine to sitting up on edge of bed	0.10	0.032	0.167	- 0.01	−0.046	0.026	Yes
4. Sitting without support	0.07	0.011	0.127	- 0.00	−0.023	0.021	No
5. Sitting to standing up	0.23	0.136	0.327	0.02	−0.009	0.045	Yes
6. Standing with support	0.12	0.049	0.199	- 0.01	−0.035	0.017	Yes
7. Standing without support	0.21	0.118	0.300	0.03	0.004	0.062	Yes
8. Standing on the non-paretic leg	0.20	0.092	0.301	0.04	−0.000	0.089	Yes
9. Standing on the paretic leg	0.18	0.061	0.297	0.02	−0.019	0.055	Yes
10. Standing, picking up a shoe from the floor	0.25	0.157	0.352	0.00	−0.024	0.026	Yes
11. Sitting down from standing up	0.16	0.074	0.237	0.02	−0.013	0.048	Yes
12. Sitting on edge of bed to supine	0.15	0.070	0.234	'-0.00	−0.032	0.030	Yes
Total score	0.31	0.219	0.402	†0.03	0.009	0.056	Yes

Abbreviations: *SwePASS*; The Swedish Postural Assessment Scale for Stroke Patients, *RP*; Relative Position, *CI*; Confidence Interval. * [12]. †Reference RP values for the total score have not been previously published.

Of the 28 patients who were referred to further rehabilitation after discharge from the stroke unit, 24 (86%) improved, 3 (11%) were unchanged and 1 (3%) deteriorated in the SwePASS score. Of those 44 patients who were assessed as not needing further rehabilitation after discharge, 29 (66%) improved, 9 (20%) were unchanged and 6 (14%) deteriorated. There was no statistically significant difference in outcome between those who were referred to training and those who were not.

### Change over time from 3 to 6 months

The results are based on the 71 patients who participated at both 3 and 6 months after stroke. At 3 months, 20 patients out of the 71 (28%) included received a maximal SwePASS score of 36. Using the total SwePASS score from 3 to 6 months after stroke, an improvement was found in postural control in 25 out of 71 patients (35%), while a deterioration was seen in 12 patients (17%) and unchanged scores in 34 patients (48%). Eighteen out of the 20 patients who received a maximum SwePASS score at 3 months also did so at 6 months. The median total SwePASS scores were 32 (min-max: 14–36) at 3 months and 33 (min-max: 15–36) at 6 months post-stroke. The mean SwePASS scores at 3 and 6 months after stroke were 32 (SD 4.2) and 32 (SD 4.0), respectively.

Table 3 shows the values of the systematic change in relative position (RP) using the SwePASS score from 3 to 6 months. Only item 8 and the total score showed a statistically significant systematic change in position. There were no significant systematic changes in concentrations of scores; however, items 8 and 9 had higher values (RC 0.05, 95% CI −0.08–0.18 and RC 0.05, 95% CI −0.08–0.17, respectively) than all of the other items and the total score (RC values from −0.02 to 0.02, 95% CI between −0.08 and 0.18). Items 2 and 3 and the total score had RC values of 0.00 (95% CI between 0.00 and 0.00). The non-systematic relative rank variance (not shown in Table 3) was also close to zero or zero for all items except item 8 (RV 0.03, 95% CI 0.000-0.062), item 9 (RV 0.02, 95% CI 0.000-0.033) and the total SwePASS score (RV 0.02, 95% CI 0.001-0.046).

**Table 3:** Change in postural control during the first year after stroke based on systematic changes in relative position of the SwePASS score

	**3 to 6 months after stroke**			**6 to 12 months after stroke**		
	**n=71**			**n=65**		
	**95% CI**			**95% CI**		
**Item**	**RP**	**Lower**	**Upper**	**RP**	**Lower**	**Upper**
1. Supine to lateral, affected side	0.03	−0.011	0.065 n.s	−0.03	−0.071	0.011 n.s
2. Supine to lateral, non-affected side	0.01	−0.033	0.061 n.s	−0.00	−0.043	0.041 n.s
3. Supine to sitting up on edge of bed	0.01	−0.013	0.041 n.s	0.00	−0.042	0.042 n.s
4. Sitting without support	0.00	*0.000	*0.000 n.s	0.02	−0.014	0.045 n.s
5. Sitting to standing up	0.01	−0.047	0.076 n.s	0.00	*0.000	*0.000 n.s
6. Standing with support	0.00	−0.037	0.038 n.s	0.00	−0.001	0.002 n.s
7. Standing without support	0.03	−0.024	0.080 n.s	0.01	−0.035	0.064 n.s
8. Standing on the non-paretic leg	0.10	0.011	0.180 sign.	−0.07	−0.140	0.006 n.s
9. Standing on the paretic leg	0.06	−0.012	0.137 n.s	−0.07	−0.143	0.009 n.s
10. Standing, picking up a shoe from the floor	0.01	−0.014	0.041 n.s	0.00	−0.014	0.044 n.s
11. Sitting down from standing up	0.03	−0.010	0.067 n.s	0.00	*0.000	*0.000 n.s
12. Sitting on edge of bed to supine	0.00	−0.000	0.001 n.s	0.01	−0.015	0.044 n.s
Total score	0.09	0.030	0.152 sign.	- 0.07	−0.134	−0.010 sign.

Abbreviations: *SwePASS*; The Swedish Postural Assessment Scale for Stroke Patients, *RP*; Relative Position, *CI*; Confidence Interval, *n.s*.; non significant and sign; significant. *Due to rounding up by the computer program, it is unclear whether the 95% CI limits of 0.000-0.000 represent values of null or very small figures.

### Change over time from 6 to 12 months

These results are based on the 65 patients who participated in both the 6 and 12-month follow-ups. At 6 months, 26 of these 65 (40%) patients received a maximal SwePASS score of 36. Using the total SwePASS score during the time period from 6 to 12 months after stroke 12 out of the 65 patients (19%) showed improved postural control while a reduction in scores was observed in 17 patients (26%) and an unchanged total score in 36 patients (55%). Of the 26 patients who received a maximum SwePASS score at 6 months, 19 also did so at 12 months. The remaining 7 patients, with a maximal SwePASS score at 6 months, deteriorated, as shown by their total SwePASS score of 34 at 12 months. The median total SwePASS score was 33 (min-max: 13–36) at 6 months and 34 (min-max: 14–36) at 12 months after stroke. The mean total SwePASS scores at 6 and 12 months after stroke were 33 (SD 4.6) and 32 (SD 4.4), respectively.

Table 3 shows the values for the systematic change in relative position (RP) using the SwePASS from 6 to 12 months after stroke. Only the total score showed a statistically significant systematic change in position. There were no significant systematic changes in the concentration of scores for the single items or the total score (RC values −0.02 to 0.09, 95% CI −0.08–0.22). Item 8 (0.01, 95% CI 0.000-0.030), item 9 (0.02, 95% CI 0.000-0.033) and the total SwePASS score (0.02, 95% CI 0.000-0.033) showed low, but significant, relative rank variance values. None of the other items showed any significant relative rank variance (RV values 0.000-0.0002, 95% CI 0.000-0.0005).

## Discussion

Our study shows that the SwePASS is responsive to change, as based on the results of assessments during the first week and 3 months after stroke, at least for those patients who do not reach a maximum score for the test at baseline. Moreover, the results showed that the patients improved in terms of postural control, as assessed using the SwePASS score, up to 6 months after stroke. We also found a slight deterioration in postural control, according to the total SwePASS score, between 6 and 12 months after stroke.

Although most of the patients improved in postural control during the first 3 months after the stroke, some patients deteriorated. The amount of training might differ, but it is unclear how much and how this influenced postural control assessed using SwePASS. The median age at inclusion in the study was 73 years, and several of the patients had other diseases and conditions that might have influenced postural control.

“Postural control” must be discussed to be able to relate the results to clinical settings. What does “*postural control*” mean and what has been estimated? We have adopted the definition of postural control of Shumway-Cock and Woollacott [27] as an integration of different sensory modalities for body position in space (stability) and different motor strategies for the choice of body movement (appropriate orientation of body segments), as well as attention processing, which constitutes the basis on which functional movement is achieved. In addition, postural control is described as emerging from an interaction of the individual with the task and the environment [27].

Our data emphasize the fact that trustworthy information about the change (a real change or not) in postural control in stroke patients can be captured using the SwePASS score. As the SwePASS score shows responsiveness to change, it is feasible for use in clinical settings. Previously published data [12,13] support this statement. Since rating scales have a fundamental role in the determination of patient care and in the evaluation of the effects of clinical interventions, besides being able to serve as an expression of clinical professionalism, these findings are of importance.

Our results concerning responsiveness are in line with the results presented by Yu *et al*. [15] and Mao *et al*. [14]. Yu *et al*. [15] found high internal responsiveness during the hospital stay in 85 patients severely disabled by stroke, with a median total PASS admission score of 16 (min 0-max 36). The time of the initial evaluation was a median of 19 days after stroke onset. Mao *et al*. [14] found a high responsiveness of PASS in 93 patients between 14 and 90 days after stroke. Compared with the mean SwePASS admission score of 28 (SD 7.1) in the present study, Mao’s population had greater stroke disabilities, with a mean total PASS admission score of 17.6 (SD 12.8). In addition, Mao *et al*. [14] had a slightly younger population with a higher percentage of cerebral haemorrhage (26%). Both Yu *et al*. [15] and Mao *et al*. [14] showed a low percentage of patients that received the maximal score (ceiling effect). Our study also showed a low ceiling effect of 14%, although there were considerably higher admission scores. However, even though we showed that the SwePASS score is responsive, our results in terms of the higher percentage of ceiling effects seen with the longer passage of time since stroke (28% at 3 months and 40% at 6 months) indicate that the SwePASS score has a reduced ability to discriminate postural control beyond 3 months after stroke. When selecting the SwePASS to estimate changes in postural control, it might not be the first choice for patients who are at the end of impairment, with mildly impaired postural control. A modification of item categories or a greater number of item categories might increase the ability of SwePASS to detect changes. This is just speculation, however.

The PASS was also shown by Benaim *et al*. [11] to be less suitable after 3 months, as based on the distribution of scores, where 38% of 58 patients had already received the maximal score 90 days after stroke.

Only item 8 and the total SwePASS score showed a significant systematic change in the positions of scores from 3 to 6 months. Our result regarding recovery does not completely correspond with that of Jørgensen *et al*. [18] who, based on a large population study, reported that recovery in Activities of Daily Living was completed within 3 months in the majority of the patients and that recovery in severely impaired patients should not been expected after 5 months. Another interesting finding was that 10 to 25% of the patients showed a deterioration in the total SwePASS score, with a higher percentage of deterioration occurring over a longer time since the onset of stroke; this result is in line with the known non-linear pattern of the function of time [19]. One possible explanation for this, perhaps in combination with older age and a natural decline in function, might be that patients receive more intensive rehabilitation interventions at the stroke unit than after discharge, with ambiguity in terms of who is responsible for continued rehabilitation intervention after discharge.

This study has some limitations. First, some patients were lost to follow-up. Offering follow-up investigations at the patients’ home would probably have decreased the number of patients lost to follow-up. Second, it would have been interesting to have several close follow-ups during the early phase, since we do not know when (especially during the first 3 months after stroke) the major part of the improvement took place. Third, more than one physiotherapist was involved in the assessments. However, we believe that any impact on the results is limited, in view of the previously published high interrater reliability [12] of the SwePASS, besides clear instructions to the physiotherapists prior to their involvement in the assessments.

Finally, we cannot differentiate between functional improvements due to spontaneous neurological recovery and improvements following rehabilitation interventions. However, in a randomized controlled trial with an intervention of 80 hours of physiotherapy vs. self-initiated exercise during the first year following stroke, Langhammer *et al*. [28] found a steady improvement in balance (as well as in Instrumental Activities of Daily Living (IADL), motor function, gait parameters and grip strength) up to 6 months after stroke, regardless of the group assignment.

Possible baseline variability may have influenced responsiveness; however, the time of the initial assessment was chosen using the hypothesis that the patients should be clinically stable (no changes due to penumbra or cerebral oedema). However, motor recovery may already have taken place during the first few days after stroke, and thus some patients may have recovered before the first assessment. Further research with larger populations and closer follow-up assessments are desirable in order to verify our findings and describe the time course of improvements in postural balance. With a focus on the significance of the one-leg standing items, it would be very interesting to investigate whether or not the SwePASS could be reduced to fewer items and still be able to give the same information.

We employed Svensson’s method [26] since it was exclusively developed to make calculations based on ordinal data and to use all the data information. Although more classical methods (Mc Nemar’s test, sign test etc.) are applicable for ordinal data, they require dichotomization of the data, which means that information from the other categories is missed. In addition, Svensson’s method makes it possible to recognize systematic disagreement caused by individual variability in assessments separately from systematic disagreement related to the group. A disadvantage in using Svensson’s method might be that changes in RP can be difficult to interpret in a clinical setting. An alternative method would be to use Rasch analysis [29], which might add value as a method of examining and comparing rating scale responsiveness [30]. Rasch analysis is a method which, if a scale is revealed to be a unidimensional scale, could provide a non-linear transformation of the scale’s ordinal raw score to interval measure. Interval data allow an accurate interpretation of change in scores and the use of parametric statistics. To improve the clinical interpretation of change in postural control, it would have been interesting to transform the values of systematic changes in position (RP) into raw SwePASS scores. This was not possible with the present data, however. One potential method could be to perform the Rasch analysis [29] on a large dataset. Finally, the responsiveness shown for the SwePASS, distribution based established, might be even more clinically meaningful if supported by person-centred responses, i.e. anchor based, using subjective experiences to answer the question of whether or not the change in postural control is important.

## Conclusions

In conclusion, the SwePASS is responsive to change. Postural control, estimated using the SwePASS score, showed an improvement during the first 6 months in patients after a first event of stroke. The measurement property in the form of responsiveness shows that the SwePASS scoring method may be considered for use in rehabilitation when assessing postural control in patients after stroke, especially during the first 3 months.

## Competing interests

The authors declare that they have no competing interests.

## Authors’ contributions

All authors made substantial contributions to the manuscript. CUP was primarily responsible for the collection of data CUP and POH were primarily responsible for the statistical analysis and interpretation of data and for drafting the manuscript. CUP and KSS were responsible for the design of the study. AE, AD and KSS critically revised the manuscript for important intellectual content. AE contributed statistical expertise. All authors read and approved the final manuscript.
